# Inhibition of ATG12-mediated autophagy by miR-214 enhances radiosensitivity in colorectal cancer

**DOI:** 10.1038/s41389-018-0028-8

**Published:** 2018-02-20

**Authors:** J. L. Hu, G. Y. He, X. L. Lan, Z. C. Zeng, J. Guan, Y. Ding, X. L. Qian, W. T. Liao, Y. Q. Ding, L. Liang

**Affiliations:** 1 Department of Pathology, Nanfang Hospital, Southern Medical University, 510515 Guangzhou, Guangdong People’s Republic of China; 2 Department of Pathology, Southern Medical University, 510515 Guangzhou, Guangdong People’s Republic of China; 3 Guangdong Province Key Laboratory of Molecular Tumor Pathology, 510515 Guangzhou, Guangdong People’s Republic of China; 4 Department of Pathology, Xinxiang Medical University, 453003 Xinxiang, Henan People’s Republic of China; 5 Department of General Surgery, Nanfang Hospital, Southern Medical University, 510515 Guangzhou, Guangdong People’s Republic of China; 6 Department of Radiotherapy, Nanfang Hospital, Southern Medical University, 510515 Guangzhou, Guangdong People’s Republic of China

## Abstract

Radioresistance hampers success in the treatment of patients with advanced colorectal cancer (CRC). Improving our understanding of the underlying mechanisms of radioresistance could increase patients’ response to irradiation (IR). MicroRNAs are a class of small RNAs involved in tumor therapy response to radiation. Here we found that miR-214 was markedly decreased in CRC cell lines and blood of CRC patients after IR exposure. Meanwhile, autophagy was enhanced in irradiated CRC cells. Mechanically, ATG12 was predicted and identified as a direct target of miR-214 by dual luciferase assay, qPCR, and Western blot. In vitro and in vivo experiments showed that miR-214 promoted radiosensitivity by inhibiting IR-induced autophagy. Restoration of ATG12 attenuated miR-214-mediated inhibition of cell growth and survival in response to IR. Importantly, miR-214 was highly expressed in radiosensitive CRC specimens and negatively correlated with plasma level of CEA. Moreover, ATG12 and LC3 expressions were increased in radioresistant CRC specimens. Our study elucidates that miR-214 promotes radiosensitivity by inhibition of ATG12-mediated autophagy in CRC. Importantly, miR-214 is a determinant of CRC irradiation response and may serve as a potential therapeutic target in CRC treatment.

## Introduction

Colorectal cancer (CRC) is the third leading cause of cancer-related deaths worldwide^1^. To date, surgical resection remains the only curative treatment that is available for CRC. Approximately 20 to 40% of CRC patients harbor a locally advanced, unresectable, non-metastatic disease termed “locally advanced CRC” at the time of diagnosis. These patients receive chemo-radiotherapy. However, due to the inherent ability of CRC to become chemotherapy and radiation resistant, the combined-modality therapy has failed to universally improve patients’ prognosis. Because radioresistance contributes significantly to challenges in the treatment of CRC, understanding the potential molecular mechanism underlying radiosensitivity or radioresistance may ultimately improve therapeutic outcomes.

MicroRNAs (miRNAs) are a class of small non-coding RNAs that regulate gene expression at the post-transcriptional level^2^. Accumulating evidence suggests strong association between deregulated miRNAs and tumor radioresistance. For example, upregulation of let-7 miRNA is related to radioresistance in human glioma cell line^3^. MiR-34 is significantly upregulated in different human cell lines after radiation and associated with radioresistance in human prostate cancer cell lines^4^. MiR-21 is related to radioresistance in a variety of cancer cell lines, including breast^5^, lung^6,7^, glioblastoma^8^, and nasopharyngeal cancers^9^. Upregulation of miR-106b^10^ and miR-100^11^ can promote radioresistance in CRC. In our previous study, we found differentially expressed miRNAs in radiated CRC cells, such as miR-622^12^ and miR-214. MiR-214, located in the chromosomal region 1q24.3, in intron 14 of the Dynamin-3 gene (DNM3), has been reported to be downregulated in several human cancers including breast cancer^13^, cervical cancer^14^, pancreatic cancer^15^, rhabdomyosarcoma^16^, and hepatocellular cancer^17^. Moreover, miR-214 modulates radiotherapy response of non-small cell lung cancer cells (NSCLC) via regulation of p38MAPK, apoptosis and senescence^18^. However, the function and mechanism of miR-214 on radioresistance in CRC remain unclear.

Autophagy is an evolutionarily conserved process that forms double-membrane autophagosome to degrade damaged organelles and unfolded proteins^19^. The formation of autophagosome is regulated by autophagy-related genes (ATGs), such as ATG12, ATG5, and microtubule-associated protein light chain 3 (LC3). ATG12 forms a conjugate complex with ATG5 and has important roles in autophagosome expansion^20^. Recent studies have shown that deregulated autophagy is associated with tumor radioresistance. Hypoxia induced accumulation of ATG5, ATG7, and ATG12 can markedly elevate autophagic activity and increase radioresistance in breast cancer cells^21^. Inhibition of ATG5 aggravates IR-induced DNA damage and apoptosis in nasopharyngeal cancer cells^22^.

In this study, we demonstrate a novel role of miR-214 in modulating the resistance of CRC cells to radiotherapy. Inhibition of ATG12-mediated autophagy by miR-214 enhances radiosensitivity. MiR-214 and ATG12 might be promising markers for the prediction of radiosensitivity in CRC patients.

## Results

### miR-214 is downregulated in response to IR

**Fig. 1:**
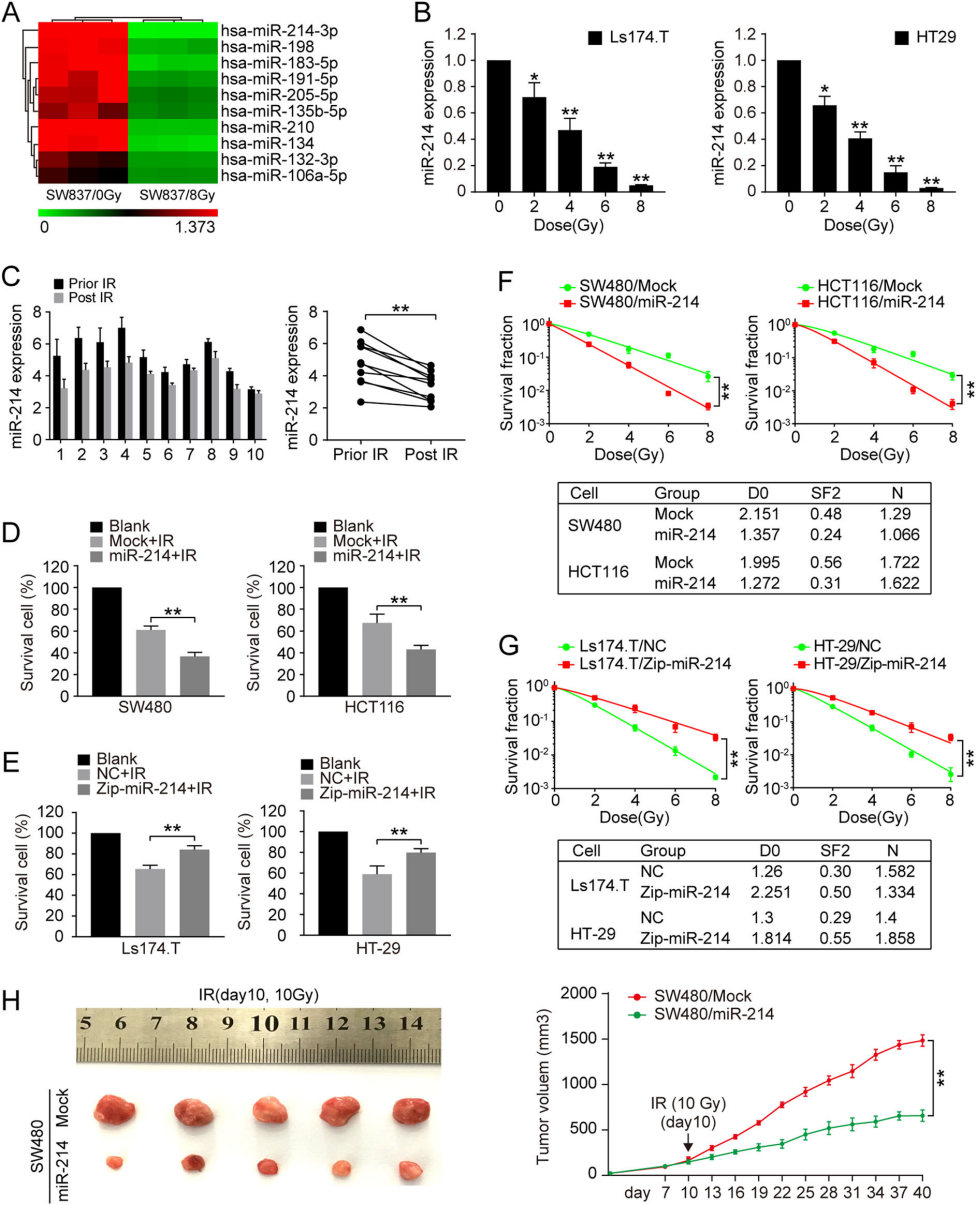
Downregulation of miR-214-induced radioresistance of CRC. **a** Differentially expressed microRNAs in SW837 cells in response to IR treatment by microRNA microarray. U6 was used as internal control. **b** MiR-214 expression in HT29 and LS174.T CRC cell lines by quantitative real-time PCR analysis. Data represent mean ± SD. *p < 0.05, **p < 0.01. **c** Plasma level of miR-214 in CRC patients after IR exposure by quantitative real-time PCR analysis. **p < 0.01. **d** Cell viability of Mock and miR-214 expressing SW480 and HCT116 cells detected by CCK-8 assay. Data represent mean ± SD. *p < 0.05, **p < 0.01. **e** Cell viability in NC and anti-miR-214 expressing Ls174.T and HT29 cells detected by CCK-8 assay. Data represent mean ± SD. *p < 0.05, **p < 0.01. **f** Clonogenic survival assay of Mock and miR-214-expressing SW480 and HCT116 cells treated by indicated irradiation dosage. Data represent mean ± SD. *p < 0.05, **p < 0.01. **g** Clonogenic survival assay of NC and miR-214-inhibiting LS174.T and HT29 cells treated by indicated irradiation dosage. Data represent mean ± SD. *p < 0.05, **p < 0.01. **h** The growth of subcutaneous tumor derived from SW480/Mock and SW480/miR-214 cells in Balb/C nude mice. Mice were treated with 10 Gy irradiation at tenth day post tumor cell injection. *N* = 5 per group, data represent mean ± SD. *p < 0.05, **p < 0.01

To identify miRNAs that regulate the IR response in CRC, a miRNA screen was performed in CRC cells treated with IR in our previous study^12^. From the list of differentially expressed miRNAs, we focused on miR-214 because it was decreased most significantly in irradiated CRC cells (Fig. 1a), and its role in IR response of CRC is unclear. We thus evaluated the association between miR-214 expression and IR. According to endogenous miR-214 expression in human CRC cell lines (Figure S1A), we chose HT29 and Ls174.T cells with high level of miR-214 to be exposed to increasing doses of IR. As shown in Fig. 1b, miR-214 expression was decreased dose-dependently in both cell lines (*p* < 0.05). Moreover, the level of miR-214 was detected in blood samples of patients with advanced CRC (*n* = 10) after radiotherapy by qRT-PCR. And miR-214 expression levels were significantly decreased in CRC patients after radiotherapy (Fig. 1c, *p* < 0.01). These data indicate that miR-214 is downregulated in CRC in response to IR.

### miR-214 increases the sensitivity to irradiation in CRC

We then explored the role of miR-214 in IR sensitivity of CRC cells. According to endogenous miR-214 expression of CRC cell lines (Figure S1A), miR-214 expressing lentivirus vector was transfected into SW480 and HCT116 cells, while miR-214-depleting lentivirus vector was transfected into Ls174.T and HT29 cells (Figure S1B, C, *p* < 0.01). CCK-8 assay showed a decreased viability in miR-214 expressing SW480 and HCT116 cells compared to control cells after IR (Fig. 1d, *p* < 0.01). Consistently, colony formation assay showed that the survival fractions of miR-214 expressing SW480 and HCT116 cells were markedly decreased after IR (Fig. 1f, *p* < 0.01). On the contrary, inhibition of miR-214 significantly increased cell viability and colony survival ability of miR-214 depleting Ls174.T and HT29 cells compared to control cells after IR (Fig. 1e, g, *p* < 0.01). To further explore the effect of miR-214 on IR response in CRC in vivo, nude mice were subcutaneously injected with SW480/Mock and SW480/miR-214 CRC cells and exposed to 10 Gy IR at 11th day post injection. As shown in Fig. 1h, subcutaneous tumors derived from SW480/miR-214 cells were significantly smaller than SW480/Mock cells (*p* < 0.01). These above findings indicate that miR-214 enhances the radiosensitivity in CRC cells.

### Radiation-induced autophagy promotes radioresistance in CRC cells

**Fig. 2:**
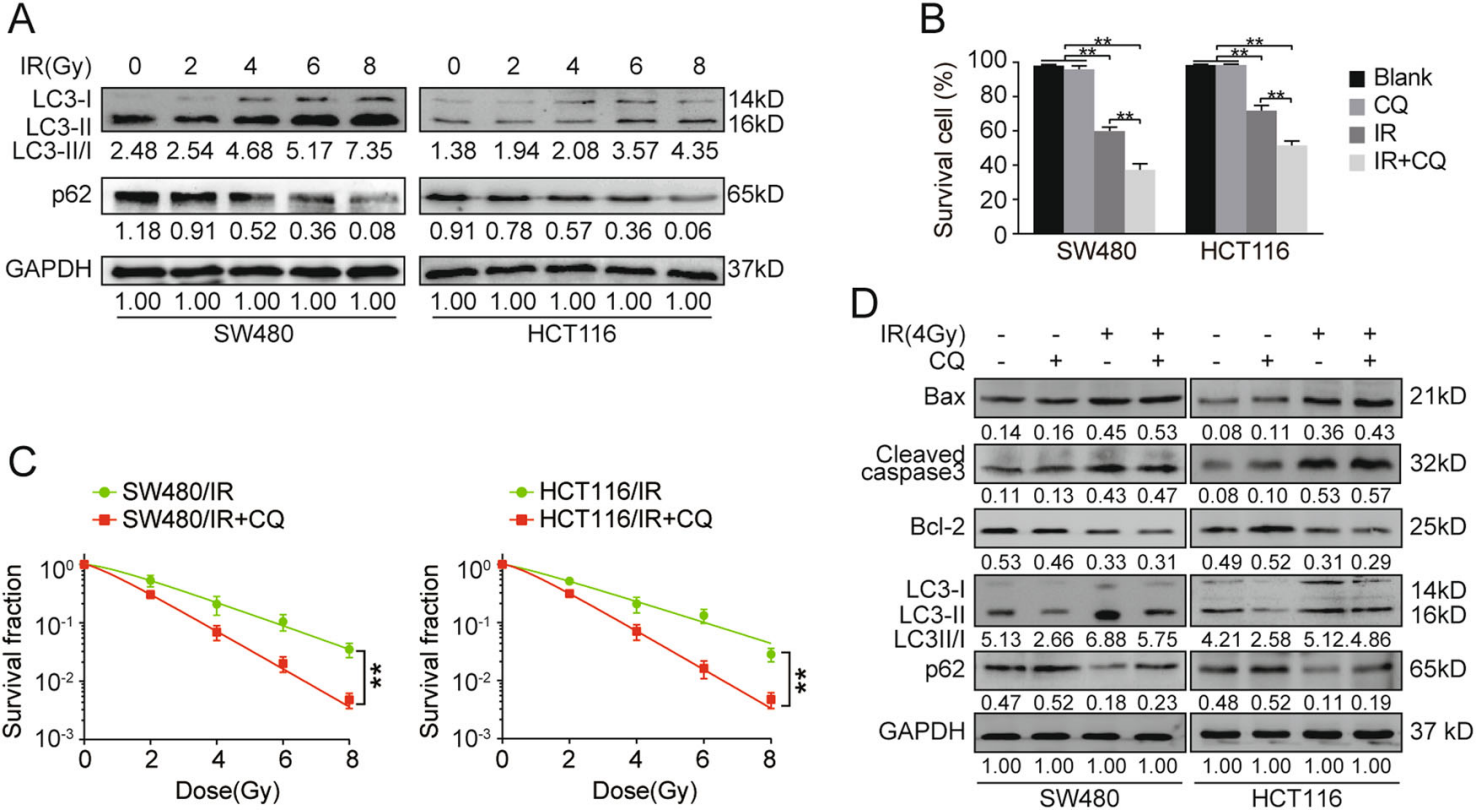
IR-induced autophagy suppressed cell apoptosis and increased radioresistance. **a** Autophagy-related proteins LC3 and p62 expression in SW480 and HCT116 cells exposed to different IR dosage by Western blot. **b** Cell viability of IR exposed SW480 and HCT116 cells treated with or without CQ by CCK-8 assay. **p < 0.01. **c** Cell survival fractions of IR exposed SW480 and HCT116 cells treated with or without CQ by plate colony formation assay. **p < 0.01. **d** Expression of Bax, cleaved caspase3, Bcl-2, LC3, and p62 in indicated SW480 and HCT116 cells by Western blot

Previous studies have found that the induction of autophagy after IR exposure contributes to tumor radioresistance through regulation of apoptosis^23–25^. We hypothesized that CRC cells may exhibit increased autophagy after IR exposure. LC3, a widely used autophagy marker, has two forms in cancer cells: the cytoplasmic form LC3-I and membrane-associated form LC3-II. LC3-I to LC3-II transition is widely used to evaluate autophagy activity^26^. As expected, we found increased level of LC3-II and reduced protein level of autophagy substrate p62 in SW480 and HCT116 cells after IR exposure compared to cells without IR exposure (Fig. 2a), which suggest radiation activated autophagy. To explore the function of IR-induced autophagy in IR response, we used chloroquine (CQ), which blocks autophagosome formation and functions as an autophagy inhibitor, to detect cell viability and clone formation ability of CRC cells. As shown in Fig. 2b, cell viability of SW480 and HCT116 cells in blank and CQ-treated group was higher than those exposed to IR. Moreover, the survival of both cells treated with IR+CQ was significantly decreased compared to those treated with IR only (*p* < 0.01). Consistent with these results, survival fractions of SW480 and HCT116 cells treated with CQ were significantly decreased compared to control cells (Fig. 2c, *p* < 0.01). We further analyzed apoptosis of CRC cells after IR exposure. Western blot results showed a decrease of anti-apoptotic protein Bcl-2 and increases of apoptotic proteins Bax and cleaved caspase3 in CQ-treated SW480 and HCT116 cells (Fig. 2d). Meanwhile, CQ decreased level of LC3-II and increased p62 level in SW480 and HCT116 cells after IR exposure. Taken together, these results indicate that IR-induced autophagy promotes radioresistance in CRC cells.

### MiR-214 inhibits CRC cell autophagy after irradiation stimulation

**Fig. 3:**
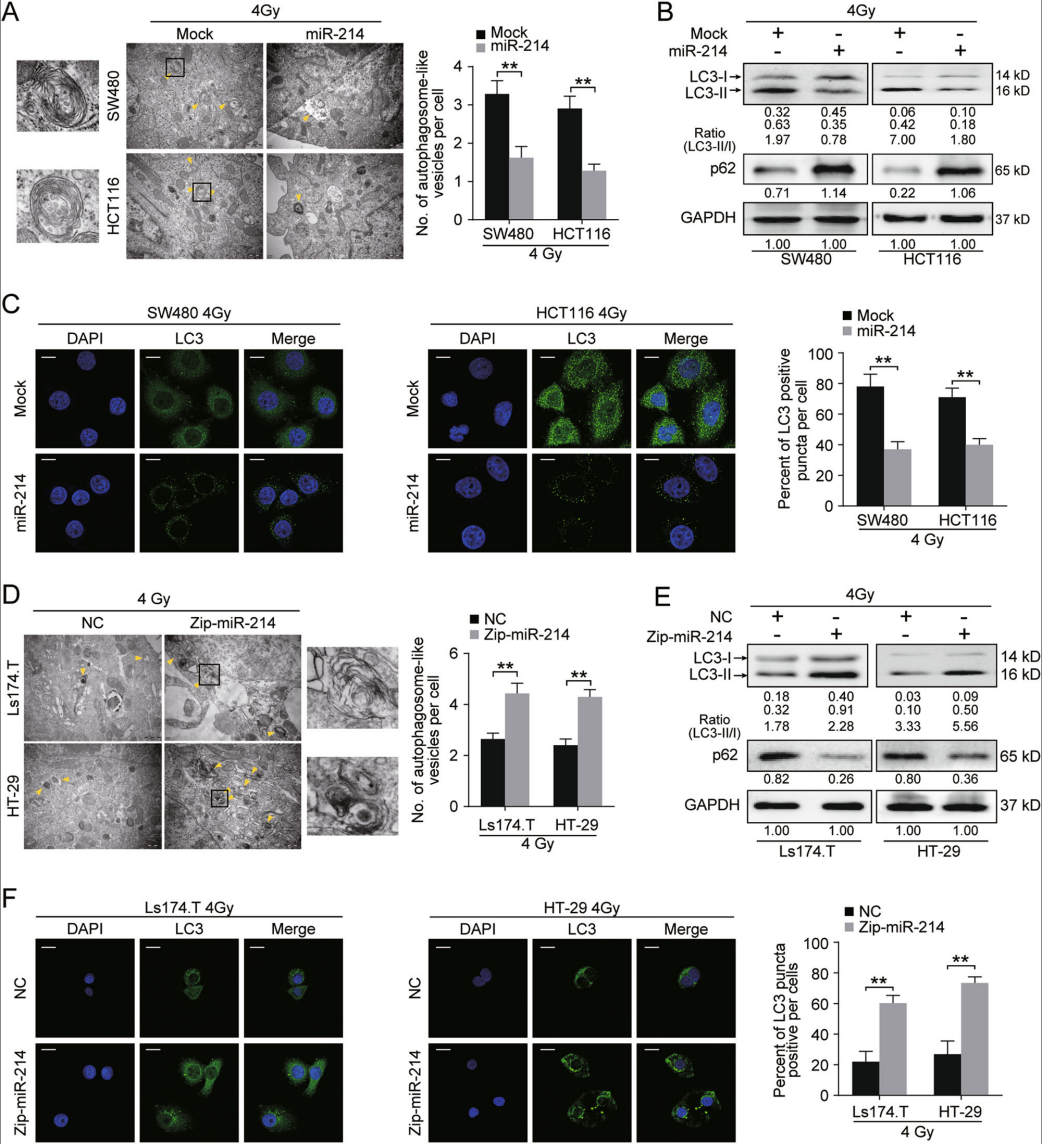
IR-induced downregulation of miR-214 was associated with increased autophagic activity in CRC cells. **a**, **d** Autophagy evaluation of SW480 and HCT116 (**a**) or Ls174.T and HT29 (**d**) cells by transmission electron microscopy (TEM). Arrow head indicated autophagosome-like vesicles induced by IR stimulation in CRC cells. Scale bar, 100 nm. The data were quantified by counting the number of autophagosome-like vesicles per cross-sectioned cell. The data are represented as mean ± SD of values obtained in three independent experiments. **p < 0.01. **b**, **e** Expression of LC3 and p62 proteins in Mock and miR-214 expressing cells (**b**) or NC and miR-214 inhibiting cells (**e**) treated by 4 Gy IR by Western blot. GAPDH was used as internal control. **c**, **f** Immunofluorescence analysis of LC3 puncta in Mock and miR-214 expressing SW480 and HCT116 cells (**c**) or NC and miR-214 inhibiting cells (**f**) treated by 4 Gy IR. The percentage of LC3 positive puncta cells was used to quantify the percentage of autophagic cells. **p < 0.01

Given that autophagy promotes radioresistance in CRC, we asked whether miR-214-induced radiosensitivity is associated with cell autophagy. Transmission electron microscope found a decreased number of autophagosome-like vesicles in miR-214 expressing SW480 and HCT116 cells after IR exposure (Fig. 3a, *p* < 0.01). Western blot showed that miR-214 significantly decreased the protein level of LC3-II in IR exposed SW480 and HCT116 cells (Fig. 3b). Consistently, immunofluorescence analysis showed a decrease in the percentage of LC3 puncta positive cells in miR-214 expressing SW480 and HCT116 cells (Fig. 3c, *p* < 0.01). In addition, the level of autophagy substrate p62 protein was increased in miR-214 expressing SW480 and HCT116 cells after IR stimulation (Fig. 3b). On the contrary, the number of autophagosome-like vesicles, LC3-II protein expression, and percentage of LC3 puncta positive cells were increased in miR-214 inhibiting LS174.T and HT29 cells compared to NC cells (Fig. 3d–f, *p* < 0.01), suggesting an increase of autophagy activity. Collectively, the above data suggest that miR-214 depresses autophagy in CRC cells after IR exposure.

### ATG12 is a bona fide target of miR-214 in CRC cells

**Fig. 4:**
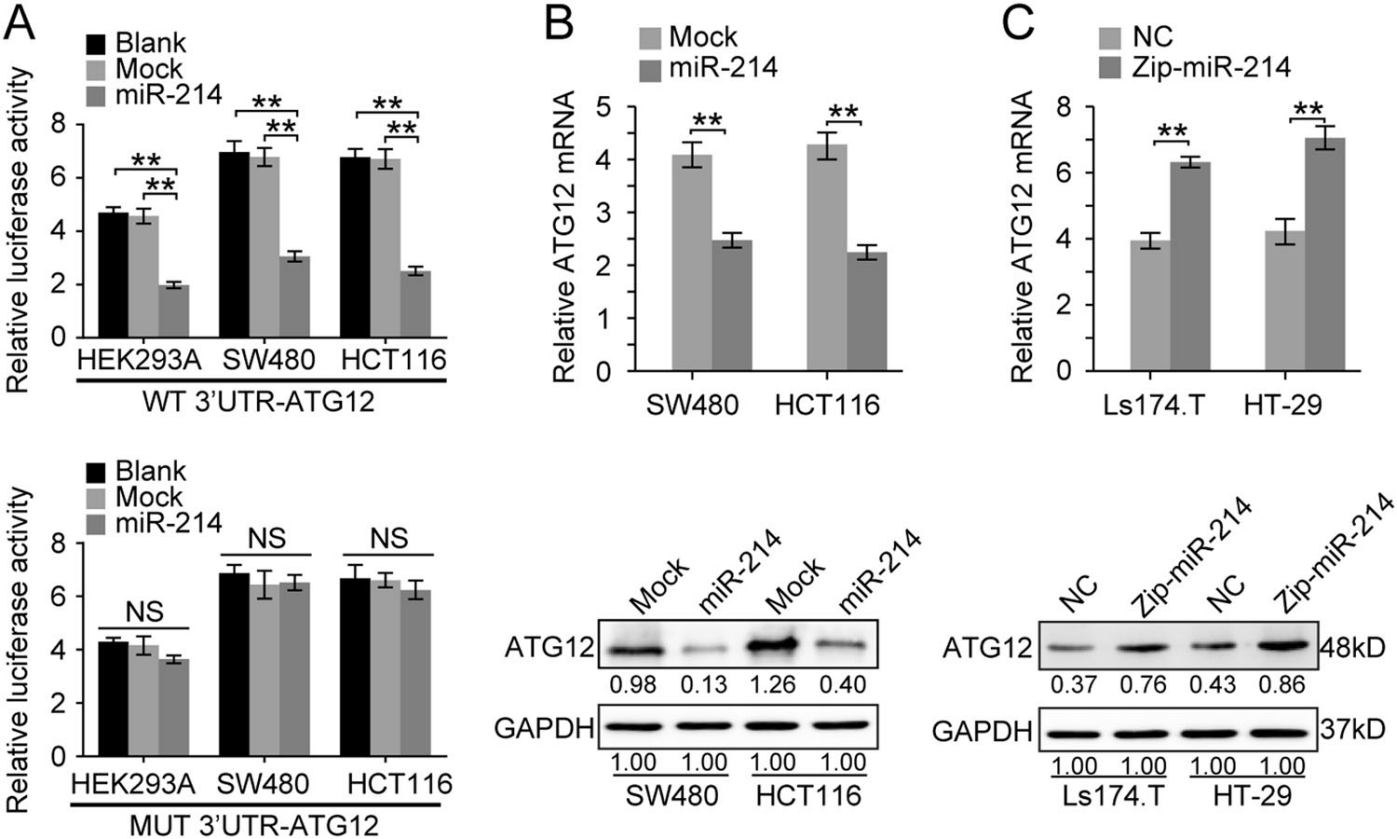
ATG12 was a downstream target of miR-214. **a** Dual luciferase activity assay of wild-type or mutated 3′UTR ATG12 in Mock and miR-214 expressing HEK293A, SW480, and HCT116 cells. Data represent means ± SD. **p < 0.01. **b** Relative expression of ATG12 in Mock and miR-214 expressing SW480 and HCT116 cells by real-time PCR and Western blot. GAPDH was used as internal control. Data represent mean ± SD. **p < 0.01. **c** Relative expression of ATG12 in NC and miR-214 inhibiting Ls174.T and HT29 cells by real-time PCR and Western blot. GAPDH was used as internal control. Data represent mean ± SD. **p < 0.01

MiRNAs bind through partial or complete sequence homology to the 3′UTR of target mRNAs and cause either block of translation or mRNA degradation. To identify potential downstream targets of miR-214, we performed a bioinformatics analysis using five miRNA databases (miRanda, TargetScan, Picta, RNAhybrid, and miRBase). Autophagy-related gene 12 (ATG12) was predicted as a target of miR-214 by all five databases. To determine whether the 3′UTR of ATG12 is a functional target of miR-214, we cloned the sequence of ATG12 3′UTR (WT-3′UTR) or the mutant sequence (MT-3′UTR) into a luciferase plasmid in HEK293A and CRC cells. As expected, the luciferase activity of ATG12 WT-3′UTR was significantly suppressed by miR-214 (Fig. 4a, *p* < 0.01), while miR-214 had no effect on ATG12 MUT-3′UTR (Fig. 4a, *p* > 0.05). qPCR and Western blot analyses showed an obvious decrease of ATG12 mRNA or protein level in miR-214 expressing cells, an increase of ATG12 level in miR-214 depleting cells (Fig. 4b, c). These data make it clear that ATG12 is a downstream target of miR-214 in CRC cells.

### ATG12 is required for miR-214-mediated CRC cell response to IR

**Fig. 5:**
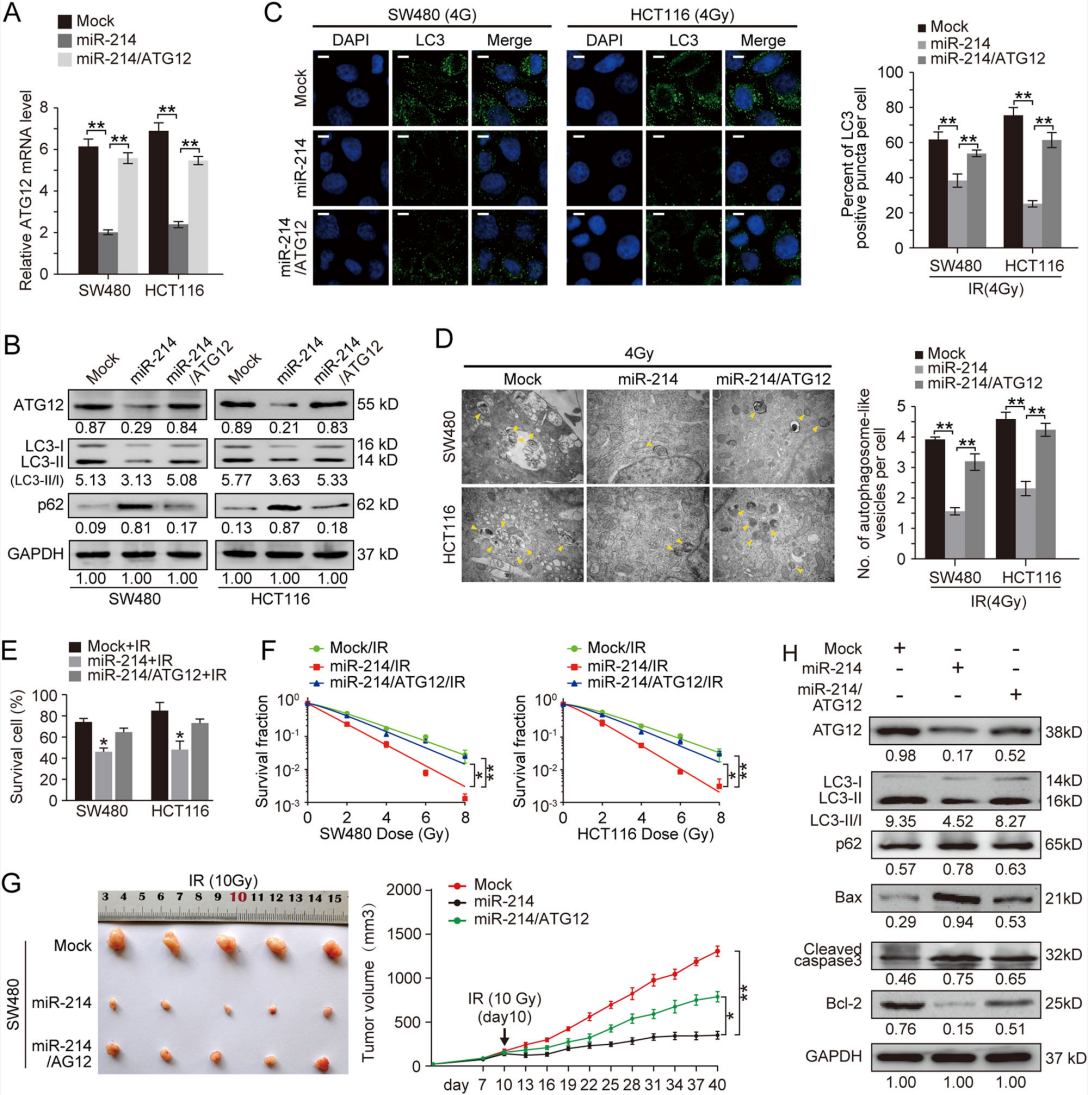
ATG12 attenuates miR-214-mediated autophagy inhibition in CRC cells. **a** Relative expression of ATG12 mRNA in Mock, miR-214 and miR-214/ATG12 SW480, and HCT116 cells by real-time PCR. Data represent mean ± SD. **p < 0.01. **b** Expression of autophagy-related proteins in Mock, miR-214, and miR-214/ATG12-expressing SW480 and HCT116 cells. **c** IF observation of LC3 puncta in Mock, miR-214, and miR-214/ATG12-expressing SW480 and HCT116 cell exposed to 4 Gy radiation. Data represent mean ± SD. **p < 0.01. **d** Autophagy evaluation of Mock, miR-214, and miR-214/ATG12-expressing SW480 and HCT116 cell exposed to 4 Gy radiation by TEM. Data represent mean ± SD. **p < 0.01. **e** Cell viability of Mock, miR-214 and miR-214/ATG12-expressing SW480 and HCT116 cells after IR exposure by CCK-8. *p < 0.05. **f** Cell survival fractions of Mock, miR-214 and miR-214/ATG12-expressing SW480 and HCT116 cells after IR exposure by plate colony formation. *p < 0.05. **p < 0.01. **g** The growth of subcutaneous tumor derived from SW480/Mock, SW480/miR-214, and SW480/miR-214/ATG12 cells in Balb/C nude mice. Mice were treated with 10 Gy irradiation at tenth day post tumor cell injection. *N* = 5 per group, data represent mean ± SD. *p < 0.05, **p < 0.01. **h** Expression of ATG12, LC3, p62 and Bax, cleaved caspase3, Bcl-2 in Mock, miR-214 or miR-214/ATG12-expressing SW480 cells-derived tumors by Western blot

To investigate whether ATG12 reverses miR-214-induced CRC cell response to IR, we performed the rescue experiments. To do so, ATG12-expressing vector without 3′UTR region was transfected into SW480/miR-214 and HCT116/miR-214 cells. The mRNA and protein levels of ATG12 were significantly decreased in miR-214 expressing SW480 and HCT116 cells (Fig. 5a, b). Re-introduction of ATG12 increased the expression of ATG12 in SW480 and HCT116 cells compared to miR-214 expressing cells (Fig. 5a, b). Moreover, restoration of ATG12 attenuated miR-214-mediated inhibition of LC3-II protein and the percentage of LC3 puncta positive cells (Fig. 5b, c). Consistently, the level of autophagy substrate p62 was decreased in miR-214/ATG12-expressing cells compared to miR-214 expressing cells after irradiation stimulation (Fig. 5b). TEM showed that ATG12 obviously alleviated the suppression of miR-214 on autophagosome-like vesicles formation in IR exposed SW480 and HCT116 cells (Fig. 5d, *p* < 0.01). In vitro function assays showed that re-introduction of ATG12 significantly reversed miR-214-mediated inhibitions on CRC cell viability and colony survival ability (Fig. 5e, f). To further demonstrate the role of ATG12 restoration in miR-214-mediated IR response, SW480/mock, SW480/miR-214, or SW480/miR-214/ATG12 cells were subcutaneously injected into nude mice. The size of tumor derived from miR-214/ATG12-expressing SW480 cells was larger than those from miR-214-expressing SW480 cells (Fig. 5g, *p* < 0.05). Moreover, tumors derived from SW480/miR-214 cells yielded significant decreases of LC3-II and Bcl-2, accompanied with increases of p62, Bax, and cleaved capase3. However, SW480/miR-214/ATG12 cells-derived tumors showed the opposite effects (Fig. 5h). These results demonstrate that miR-214 enhances radiosensitivity through ATG12-mediated autophagy in CRC.

### MiR-214 is a prediction marker for CRC radiotherapy

**Fig. 6:**
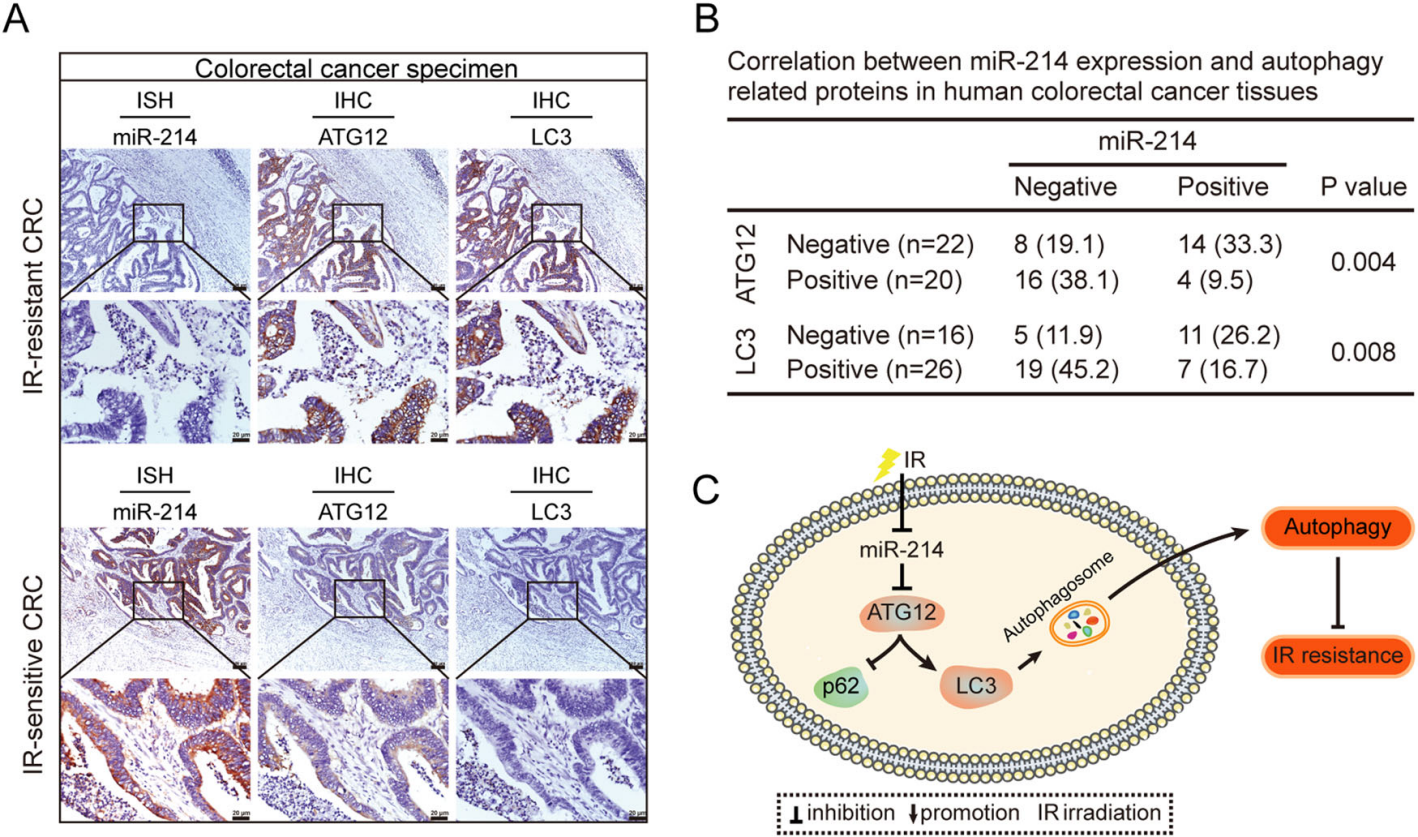
Correlations between miR-214 and ATG12, LC3 in human CRC tissues. **a** In situ hybridization (ISH) and immunohistochemistry analysis (IHC) of miR-214, ATG12, and LC3 in continuous sections of paraffin-embedded human CRC specimen. Bars represent 50 μm. Representative images were shown. **b** Correlations between miR-214 expression and ATG12 and LC3 in forty-two cases of human CRC tissues. **c** Schematic of miR-214 in the regulation of cell response to irradiation. Briefly, miR-214 inhibits cell autophagy by directly targeting ATG12. IR exposure enhances cell autophagy and inhibits apoptosis through downregulation of miR-214, thus causing IR resistance

Forty-two CRC participants received standard dose radiation therapy followed by TME surgery were enrolled in this study. Pathologic responses were scored according to the Mandard TRG scale by two independent pathologists. Results of ISH assay showed that 15/26 CRC patients defined as complete response or partial response (TRGI~III) to IR were miR-214 positive, while 13/16 CRC patients defined as stable or progressive disease were miR-214 negative (Table S2). miR-214 expression was higher in radiosensitive CRC tissues than radioresistant ones (Fig. 6a). Clinicopathologic analyses showed that patients with low level of miR-214 were accompanied with a higher CEA level (Table S2, *p* = 0.019). The expression correlations among miR-214, ATG12, and LC3 were also explored in the same CRC tissues. In contrast with the expression pattern of miR-214 in CRC tissues, negative or very weak of ATG12 and LC3 levels were observed in radiosensitive CRC tissues (Fig. 6a). Spearman analyses showed that miR-214 was negatively correlated with ATG12 and LC3 in clinical CRC tissues, respectively (Fig. 6b, *p* < 0.01). These data reveal the potential of miR-214 as a biomarker for CRC patients to predict their response to radiation treatment.

## Discussion

Increasing evidence show that miRNA expression patterns are involved in predictions and modifications of anticancer treatments. The relationship between miRNAs and tumor sensitivity to radiotherapy has been reported in different cancers. Upregulation of miR-668 markedly enhances the radioresistance of human breast cancer cells via targeting IkBα^27^. MiRNA-558 promotes radioresistance of human lung cancer cells by targeting apoptosis-associated tyrosine kinase^28^. MiR-19b-3p enhances radioresistance of nasopharyngeal cancer radiosensitivity by targeting TNFAIP3/NF-κB axis^29^. In our previous studies, a list of deregulated miRNAs was screened out using miRNA microarray, such as miR-424, miR-622, and miR-214. MiR-424 has been reported to enhance radiosensitivity of cervical cancer cells by targeting aprataxin^30^. MiR-622 is increased after IR exposure and induces radioresistance of CRC cells by downregulation of Rb^12^. In this study, miR-214 was a most downregulated miRNA in IR exposed CRC cells, but the role of miR-214 in CRC radioresistance is still elusive. We found that miR-214 was apparently downregulated in human CRC cells after IR exposure. Moreover, a reduced level of miR-214 was seen in peripheral blood of patients with CRC after IR therapy. In vitro and in vivo experiments showed that miR-214 sensitized CRC cells to IR. Our findings demonstrate miR-214 as a potential sensitizer to IR in CRC, which is against the radioresistant role of miR-214 in non-small cell lung cancer^18^. The possible reason may be that miR-214 functions as tumor suppressor or oncomiRNA in different tumors^13,14,31^. Thus further researches are needed to explore the underlying mechanisms.

Previous studies have demonstrated the functions of miRNA-regulated autophagy in IR response of cancer cells. MiR-122 and miR-144 target PIM1 to inhibit cell autophagy and enhance radiosensitivity in prostate cancer cells^32^. MiR-205 suppresses autophagy and enhances radiosensitivity of prostate cancer cells through downregulation of TP53INP1^33^. MiR-32 promotes radioresistance by targeting DAB2IP and regulating autophagy in prostate cancer cells^34^. In our study, we found an induction of autophagy in SW480 and HCT116 cells after IR exposure. Inhibition of autophagy by CQ promoted cell radiosensitivity via induction of apoptosis, suggesting cell protective role of autophagy in response to IR. Moreover, we found that miR-214 could markedly suppress cell proliferation and survival ability by inhibiting autophagy in response to IR. Therefore, miR-214-mediated inhibition of autophagy might be a potential strategy to enhance radiosensitivity of CRC.

Next, we explored the underlying mechanism of miR-214-mediated inhibition of autophagy in CRC. Using five different online databases, we screened ATG12 as a target of miR-214. ATG12 is an important protein in the elongation of autophagosome^35^. We confirmed the binding between miR-214 and ATG12 3′UTR and the regulative effect of miR-214 on ATG12 expression. Restoration of ATG12 attenuated miR-214-mediated inhibition of cell proliferation and colony survival ability in IR exposed cells. Moreover, ATG12 markedly increased the volume of subcutaneous tumor, accompanied with increased levels of LC3-II, Bcl-2 and decreased levels of Bax and cleaved caspase3. These data reveal that miR-214 inhibits ATG12-induced autophagy and increases cell apoptosis, thus leading to an increase of radiosensitivity in response to IR.

Finally, we analyzed the relationship between miR-214 expression and radiosensitivity in CRC tissues. Emerging studies showed that miR-214 was deregulated in many human tumor, including breast, ovarian, gastric, and colon cancer^31,36–38^. Our results showed that miR-214 expression was positively related to complete IR response in CRC patients, negatively related to CEA level in blood, suggesting a potential role in predicting the efficacy of radiotherapy in CRC patients. Previous study reported that ATG12 was associated with radioresistance of pancreatic cancer cells. Consistently, we found that ATG12 was highly expressed in radioresistant CRC specimens and negatively associated with miR-214. Upregulation of miR-214 and downregulation of ATG12 might function well to predict the IR response of CRC patients.

To conclude, our study characterizes a mechanism of anti-radioresistance role of miR-214 by inhibiting autophagy (Fig. 6c). Our data provide evidence that miR-214 significantly increases radiosensitivity of CRC via inhibition of autophagy and induction of apoptosis. MiR-214 achieves the anti-radioresistant effect by targeting ATG12, a key autophagy-related gene. Importantly, miR-214 expression predicts sensitive response to IR. Thus, targeting miR-214 might offer a potential therapeutic target for CRC radiotherapy.

## Materials and methods

### Cell lines

Human CRC cell lines SW837, LoVo, SW620, SW480, HCT116, HT29, LS174.T, and human embryonal kidney 293 cells were purchased from American Type Culture Collection. All cells were maintained in RPMI1640 medium (GIBCO, Gaithersburg, MD, USA) supplemented with 10% fetal bovine serum (FBS, HyClone, Logan, USA) l-glutamine (2 mM), penicillin (100 U/ml), and streptomycin (100 mg/ml, Gibco, Invitrogen, Paisley, UK) at 37 °C and 5% CO_2_.

### Patients and clinical specimens

Human CRC samples were collected from 42 patients after surgical resection at Nanfang Hospital, Southern Medical University (Guangzhou, China) between January 2011 and December 2015. Informed consent was obtained from patients, and the study was approved by the committees for the ethical review of research at Southern Medical University. All resected samples were histologically examined by HE staining. Formalin-fixed, paraffin-embedded samples were used to detect the expressions of miR-214, ATG12, and LC3.

### Construction of plasmids and transfection

Lentiviral construct expressing miR-214 (Lenti-miR microRNA precursor clone collection; System Biosciences) or repressing miR-214 (miRZips lentiviral-based microRNA inhibition; System Biosciences) was packaged using the pPACKH1 lenti-vector Packaging Kit (System Biosciences). Lentiviral constructs were used to transfect CRC cells to establish cells stably expressing or repressing miR-214. In the rescue experiments, miR-214/ATG12 co-expressing cell was constructed by transfecting ATG12 lentivirus expressing vector into miR-214 expressing cells.

### Irradiation and clonogenic assays

After 48 h of transfection, cells were transferred to complete medium and seeded on 6-well plates in triplicate and exposed to radiation at the doses indicated using a 6-MV X-ray generated by a linear accelerator (Varian 2300EX at a dose rate of 5 Gy/min; Varian MedicalSystems, Palo Alto, CA). After incubation at 37 °C for 14 days, cells were fixed in 100% methanol and stained with 1% crystal violet. Colonies containing >50 cells were counted under a light microscope (Olympus, Japan). The surviving fraction was calculated as previously described^12^. Each assay was repeated in three independent experiments.

### Quantitative real-time PCR

Total RNA was extracted from cells using Trizol reagent (Invitrogen, USA). MiR-214 and RNU6B were detected by using All-in-one miRNA kit (GeneCopeia, Guangzhou, China). First-strand cDNA was synthesized using the PrimeScript RT Reagent kit (Perfect Real Time, Takara, Dalian, China) and amplified for the detection of miR-214, ATG12, and GAPDH using primers listed in supplementary Table 1. The relative expression levels were calculated using the 2-comparative Ct (2^−ΔΔCt^) method. MiRNA expression levels were normalized for RNU6B, and mRNA expression levels were normalized for GAPDH.

### CCK-8 assay

Cell viability was detected using Cell Counting Kit-8 (CCK-8) assay as followed. Briefly, 96-well plates were seeded with 1 × 10^3^ cells per well with 100 μl cell culture medium and grew overnight. 10 μl CCK-8 reagent was then added into each well and the optical absorbance value (Optical Density value, OD value) of reading was analyzed after incubation for 2 h at 37 °C and 5% CO_2_. The absorbance value of each well in the subsequent 7 days was measured by microplate reader at wavelength of 450 nm. Each assay was repeated in three independent experiments.

### Luciferase activity assay

For 3′UTR luciferase reporter assays, the 3′UTR segment of ATG12 containing putative miR-214 binding site was amplified by PCR and inserted into the vector. A mutant construct in miR-214 binding sites of ATG12 3′UTR region was also generated using Quick Change Site-Directed Mutagenesis Kit (Angilent). Co-transfections of ATG12 3′UTR plasmid with miR-214 lentivirus vector into the cells were accomplished by using Lipofectamine 2000 (Invitrogen). Luciferase activity was measured 48 h after transfection by the Dual-Luciferase Reporter Assay System (Promega). Each assay was repeated in three independent experiments.

### Xenograft studies

All animal experiments were conducted in strict accordance with the principles and procedures approved by the Committee on the Ethics of Animal Experiments of Southern Medical University. Nude mice were randomized divided into different groups using random number generator. For xenograft tumor assays, 4 × 10^6^ cells treated as indicated were injected subcutaneously into the back of 4- to 6-week-old nude mice (*n* = 6 per group). When the tumor volume reached 200 mm^3^, it was irradiated with a single 10-Gy dose 10 days after the injection. Tumor size was calculated every 2 days using the following formula: (length × width^2^)/2. The average volume for each group was obtained. Protein was extracted from the tumor lumps treated as described.

### In situ hybridization

Briefly, 3-μm sections were deparaffinized, rehydrated, and digested. Sections were then reconstituted with hybridization solution and incubated at 41 °C for 18 h with digoxigenin-labeled nucleic acid modified probes (Hongtu, Guangzhou, China). The slides were then blocked and incubated with an anti-digoxigenin antibody (Boshide, Wuhan, China) at 37 °C for 1 h. The visualization signal was developed with 3,3-diaminobenzidine tetra hydrochloride staining, and the slides were counterstained in hematoxylin and observed under a light microscope (Olympus, Japan). The slides were then independently scored by two pathologists as: negative (−), positive (+ to ++).

### Immunohistochemical staining

Immunohistochemical staining was conducted using Immunohistochemistry Kit (Maixin, China). Briefly, 4 μm-thick histology sections were deparaffinized and hydrated. Antigen retrieval was performed by boiling at 100 °C for 10min in 10 mmol/l citrate buffer (pH = 6.0). Then sections were incubated with polyclonal antibody against human ATG12 (Abcam, Cambridge, UK), LC3 (Abcam, Cambridge, UK) overnight at 4 °C. Sections were then incubated with horseradish-peroxidase-conjugated anti-goat secondary antibody (DakoCytomation, Glostrup, Denmark) for 1 h at room temperature. The visualization signal was developed with 3,3-diaminobenzidine tetra hydrochloride staining, and the slides were counterstained in hematoxylin and observed under a light microscope (Olympus, Japan). For staining of ATG12 and LC3, the scores of positively labeled cell were recorded as: 0 (no staining, 0%), 1 (staining range, 1–25%), 2 (staining range, 26–50%), 3 (staining range, 51–75%) or 4 (staining range, 76–100%). Intensity scores were recorded as: 0, no labeling; 1, weak labeling; 2, moderate labeling; or 3, strong labeling. The multiplication of the above two scores representing the results as follows: 0–4 represents low expression, 5–12 represents high expression.

### Statistical analysis

All statistical analyses were performed using SPSS 13.0 statistical software and were calculated from three independent experiments. Statistical significance was determined using two-tailed Student *t* test, Fisher’s exact test, or one-way analysis of variance (ANOVA) as appropriate. Pearson’s or Spearman’s correlation coefficient was used to measure the degree of the linear relationship of gene expression levels. *p* < 0.05 was considered to be statistically significant.

## Electronic supplementary material

Supplemental Figure S1

Supplemental figure legend

Supplemental table
