# Supplementary figures and images for: Inhibition of ATG12-mediated autophagy by miR-214 enhances radiosensitivity in colorectal cancer
Source: Oncogenesis. 2018 Feb 20;7(2):16. doi: 10.1038/s41389-018-0028-8 (PMC5833763; doi:10.1038/s41389-018-0028-8)

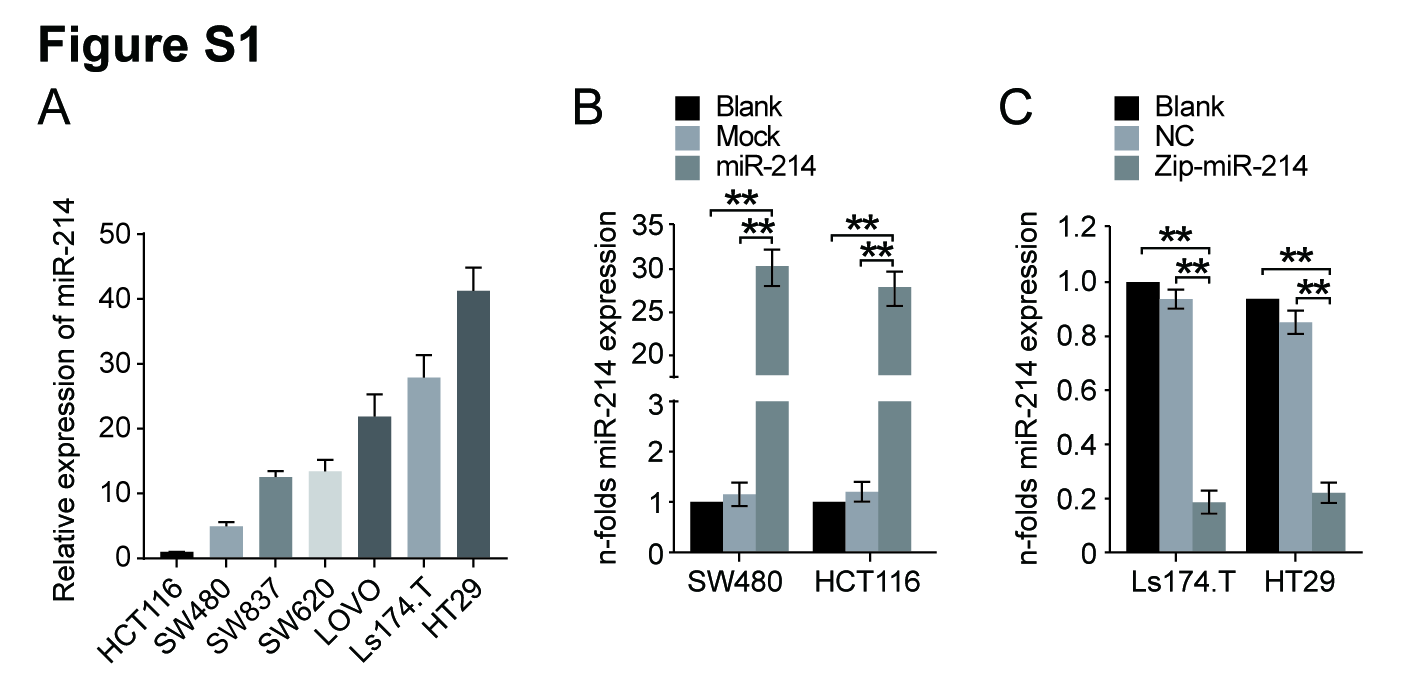

Supplement: Supplementary file 1 — Supplemental Figure S1 [file 41389_2018_28_MOESM1_ESM.tif]
