# Supplementary material for: Inhibition of ATG12-mediated autophagy by miR-214 enhances radiosensitivity in colorectal cancer
Source: Oncogenesis. 2018 Feb 20;7(2):16. doi: 10.1038/s41389-018-0028-8 (PMC5833763; doi:10.1038/s41389-018-0028-8)
Supplement: Supplementary file 2 — Supplemental figure legend [file 41389_2018_28_MOESM2_ESM.doc]

**Figure S1 Confirmation of transfection efficiency and cell response to IR in CRC cells.**

1. Endogenous miR-214 expression in human CRC cell lines. Data represents mean ± sd. All experiments were triplicate repeated.
2. Expression of miR-214 in Blank, NC, and miR-214-inhibiting Ls174.T and HT29 cells. Data represents mean ± sd. ** means p<0.01. All experiments were triplicate repeated.
3. Expression of miR-214 in Blank, Mock, and miR-214 expressing SW480 and SW620 cells. Data represents mean ± sd. ** means p<0.01. All experiments were triplicate repeated.
