# Supplementary material for: Inhibition of ATG12-mediated autophagy by miR-214 enhances radiosensitivity in colorectal cancer
Source: Oncogenesis. 2018 Feb 20;7(2):16. doi: 10.1038/s41389-018-0028-8 (PMC5833763; doi:10.1038/s41389-018-0028-8)
Supplement: Supplementary file 3 — Supplemental table [file 41389_2018_28_MOESM3_ESM.docx]

Table S1 Sequence of primer for qRT-PCR

| Gene | Primer Sequence |
| --- | --- |
| MiR-214 forward | 5’-AGCATAATACAGCAGGCACAGAC-3’ |
| MiR-214 reverse | 5’-AAAGGTTGTTCTCCACTCTCTCAC-3’ |
| ATG12 forward | 5′-TTTGCTAAAGGCTGTGGG-3′ |
| ATG12 reverse | 5′-AAGGAGCAAAGGACTGAT-3′v |
| GAPDH forward | 5′-GGGAGCCAAAAGGGTCATCATCTC-3′ |
| GAPDH reverse | 5′-CCATGCCAGTGAGCTTCCCGTTC-3′ |

Table S2. Clinicopathologic significance of miR-214, ATG12, and LC3 expression in CRC^1^ patients

| **Features** | **N=42** | **miR-214** | | **χ^2^** | ***P*** | **ATG12** | | **χ^2^** | ***P*** | **LC3** | | **χ^2^** | ***P*** |
| --- | --- | --- | --- | --- | --- | --- | --- | --- | --- | --- | --- | --- | --- |
|  |  | **Negative** | **Positive** |  |  | **Negative** | **Positive** |  |  | **Negative** | **Positive** |  |  |
| **Age** |  |  |  |  |  |  |  |  |  |  |  |  |  |
| ≤50 years | 21 | 13 | 8 | 0.389 | 0.533 | 12 | 9 | 0.382 | 0.537 | 10 | 11 | 1.615 | 0.204 |
| >50 years | 21 | 11 | 10 |  |  | 10 | 11 |  |  | 6 | 15 |  |  |
| **Gender** |  |  |  |  |  |  |  |  |  |  |  |  |  |
| Male | 33 | 20 | 13 | 0.754 | 0.385 | 19 | 14 | 1.666 | 0.197 | 12 | 21 | 0.196 | 0.658 |
| Female | 9 | 4 | 5 |  |  | 3 | 6 |  |  | 4 | 5 |  |  |
| **CEA** |  |  |  |  |  |  |  |  |  |  |  |  |  |
| <5 ug/L | 24 | 10 | 14 | 5.477 | **0.019** | 11 | 13 | 0.963 | 0.327 | 10 | 14 | 0.303 | 0.582 |
| ≥5 ug/L | 18 | 14 | 4 |  |  | 11 | 7 |  |  | 6 | 12 |  |  |
| **CA199** |  |  |  |  |  |  |  |  |  |  |  |  |  |
| <37 U/ml | 29 | 14 | 15 | 3.008 | 0.083 | 17 | 12 | 1.462 | 0.227 | 13 | 16 | 1.801 | 0.180 |
| ≥37 U/ml | 13 | 10 | 3 |  |  | 5 | 8 |  |  | 3 | 10 |  |  |
| **TRG scale^2^** |  |  |  |  |  |  |  |  |  |  |  |  |  |
| I~III^3^ | 26 | 11 | 15 | 6.133 | **0.013** | 17 | 9 | 4.627 | **0.031** | 13 | 13 | 4.102 | **0.043** |
| IV^4^ | 16 | 13 | 3 |  |  | 5 | 11 |  |  | 3 | 13 |  |  |

^1.^ CRC, colorectal cancer;

^2.^ TRG scale

^3.^ TRG scale I~III, complete or partial response;

^4.^ TRG scale IV, CRC progression.
